# Simultaneous determination of transient free radicals and reaction kinetics by high-resolution time-resolved dual-comb spectroscopy

**DOI:** 10.1038/s42004-020-00353-6

**Published:** 2020-07-30

**Authors:** Pei-Ling Luo, Er-Chien Horng

**Affiliations:** Institute of Atomic and Molecular Sciences, Academia Sinica, Taipei, 10617 Taiwan

**Keywords:** Atmospheric chemistry, Infrared spectroscopy, Chemical physics

## Abstract

Quantitative determination of multiple transient species is critical in investigating reaction mechanisms and kinetics under various conditions. Dual-comb spectroscopy, a comb-laser-based multi-heterodyne interferometric technique that enables simultaneous achievement of broadband, high-resolution, and rapid spectral acquisition, opens a new era of time-resolved spectroscopic measurements. Employing an electro-optic dual-comb spectrometer with central wavelength near 3 µm coupled with a Herriott multipass absorption cell, here we demonstrate simultaneous determination of multiple species, including methanol, formaldehyde, HO_2_ and OH radicals, and investigate the reaction kinetics. In addition to quantitative spectral analyses of high-resolution and tens of microsecond time-resolved spectra recorded upon flash photolysis of precursor mixtures, we determine a rate coefficient of the HO_2_ + NO reaction by directly detecting both HO_2_ and OH radicals. Our approach exhibits potential in discovering reactive intermediates and exploring complex reaction mechanisms, especially those of radical-radical reactions.

## Abstract (web summary)

The reaction of HO_2_ radicals with NO is a critical part of the atmospheric ozone cycle, but the plethora of involved species makes it hard to fully decipher. Here, quantitative and simultaneous determination of multiple reaction species, including HO_2_ and OH radicals, is achieved by means of high-resolution time-resolved dual-comb spectroscopy.

## Introduction

Time-resolved infrared spectroscopy plays an essential role in atmospheric, biological, and combustion chemistry studies, providing both temporal and spectral resolution for the identification of unstable molecules with unique vibrational spectral features and the observation of time-dependent spectral variations of multispecies reaction processes. In the molecular fingerprint region, the step-scan Fourier transform infrared (ss-FTIR) spectrometer^1^ has been recognized as a powerful tool with broadband spectral measurement capability, which allows it to record several vibrational bands simultaneously and thus provide decisive information for molecular identification^2,3^. However, the conventional ss-FTIR spectrometer requires extremely long measurement times for acquiring a time-resolved spectrum with adequate spectral resolution and sensitivity, making it difficult to use for kinetic studies. By contrast, sensitive detection of transient species can be achieved by using infrared continuous wave (cw) lasers coupled with the multipass absorption cell or wavelength modulation technique^4,5^. Although multispecies monitoring may be implemented by coupling several cw lasers into a system, each probe only provides the time-dependent absorption signals at a single wavelength. Hence, it is still challenging to study complex reaction system and ensure probing the targeted absorption lines without any interference from other species under various conditions.

In recent years, direct frequency comb spectroscopy (DFCS) techniques^6–11^, which enable high resolution, rapid, and broadband measurements, have been developed for different spectral regions and used in various applications. Time-resolved spectroscopy based on DFCS methods has been used for monitoring spectral variations under flash photolysis^12^, electric discharge^13^, laser-induced plasma^14,15^, and combustion conditions^16^ as well as for studying gas-phase reaction kinetics^17–19^, protein dynamics^20^, and population relaxation processes^21^. In particular, time-resolved DFCS methods can be demonstrated in the mid-infrared (MIR) region to achieve sensitive molecular identification and quantitation^12,13,17–20^. For instance, a MIR virtually imaged phased array (VIPA) spectrometer with a spectral coverage of 65 cm^−1^ and an optical resolution of 1 GHz (approximately 0.033 cm^−1^) has been employed for studying the OD + CO reaction and recording the vibrational spectra of DOCO radicals^12,17,18^. In that scheme, the temporal resolution can be set down to tens of microseconds; however, it may be limited by the camera integration time. In addition, the spectral resolution of approximately 0.03 cm^−1^ is restricted by the properties of elements, including the VIPA etalon, grating and camera, inside the spectrometer. Two identical femtosecond optical parametric oscillator combs with a spectral region of 3.15−3.45 μm has been used to observe the time-dependent phenomena of a CH_4_/He gas mixture under discharge^13^. A spectral resolution of 6 GHz (0.2 cm^−1^) and a temporal resolution of 20 μs can be achieved through the conventional Fourier transformation. However, the mode-locked-laser-based MIR dual-comb spectrometer is relatively complex and difficult to operate. A quantum cascade laser (QCL) based dual-comb spectrometer (QCL-DCS) with a spectral coverage of 55 cm^−1^ around 8.2 μm has been used for studying the single-shot spectra and kinetics of protein reactions^20^. The dual-comb spectra were recorded with a spectral resolution of a few wavenumbers at sub-microsecond time resolution and compared with the ss-FTIR spectra. The QCL-DCS has been presented with a compact system;^19,20^ nevertheless, it may be more suitable for time-resolved measurements at relatively low spectral resolution due to the large comb mode spacing (approximately 0.3 cm^−1^) of QCLs.

To investigate rotationally resolved spectra and study gas-phase reaction kinetics with distinguishing probes of multiple species, the spectral resolution of the time-resolved spectrometer must be higher than the observed absorption line width. In the 3-μm spectral region, the absorption line width of most molecules is typically a few hundred MHz at low pressure (<100 Torr) and room temperature, and the absorption line width depends on the effects of Doppler and pressure broadenings. In time-resolved dual-comb spectroscopy, a trade-off exists among the spectral resolution, temporal resolution, and spectral coverage of the frequency combs. Although dual-comb system with mode-locked or quantum cascade lasers can offer a wide measurement range without wavelength sweep, the system is unable to achieve high spectral resolution (<0.01 cm^−1^) at adequate temporal resolution (tens of microseconds) for simultaneously distinguishing multiple reaction intermediates.

In this article, we report high-resolution time-resolved dual-comb spectroscopy for simultaneous determination of multiple reaction species and kinetic studies of the HO_2_ + NO reaction, which is one of the most important reactions in atmospheric chemistry^22,23^. The reaction of HO_2_ radicals with NO, leading to the formation of OH radicals and NO_2_, plays a critical role in the O_3_ formation cycle^24,25^. To investigate the HO_2_ + NO reaction, we first record the time-resolved spectra of the HO_2_ radicals near 3 μm by employing an electro-optic dual-comb spectrometer coupled with a Herriott reaction cell upon ultraviolet photolysis of the precursor mixtures. Multiple reaction species, including the precursor (methanol, CH_3_OH), the stable product (formaldehyde, H_2_CO), and HO_2_ radicals, are observed and distinguished based on rotationally resolved spectra and distinct temporal profiles. Moreover, simultaneous measurements of CH_3_OH, HO_2_, H_2_CO, and OH signals are achieved by recording the time-resolved spectra at around 3422 cm^−1^. Hence, the kinetics of the HO_2_ + NO reaction can be studied through the direct detection of both HO_2_ and OH radicals and the rate coefficient of the HO_2_ + NO reaction can be derived by model fitting of the temporal absorbance profiles of HO_2_ and OH radicals under different experimental conditions.

## Results

### High-resolution time-resolved dual-comb spectroscopy

**Fig. 1:**
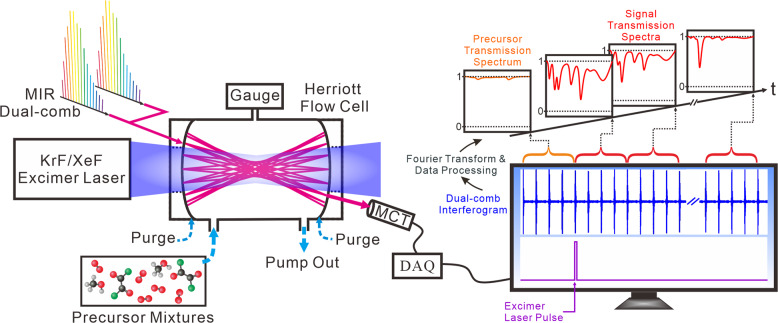
Schematic of high-resolution time-resolved dual-comb spectroscopy. Two mid-infrared (MIR) combs with slightly different repetition rates were combined and sent to the multi-pass Herriott cell. After passing through the Herriott cell, the dual-comb beam was detected with a photovoltaic HgCdTe (MCT) detector and digitized using a data acquisition board (DAQ). An excimer laser was used for laser photolysis of the precursor mixtures to generate radicals. After Fourier transformation of the time-dependent dual-comb interferograms and data processing, a precursor transmission spectrum before photolysis and time-resolved signal transmission spectra after photolysis were obtained.

The high-resolution time-resolved absorption spectroscopy was performed by means of an electro-optic dual-comb system coupled with a Herriott flow cell, as illustrated in Fig. 1. Our MIR dual-comb system was constructed using a pair of electro-optic combs at 1050 nm and a tunable cw laser (765−800 nm) with difference frequency generation (DFG)^26^. An excimer laser operating at 1 Hz was used for the flash photolysis of the precursor mixtures to generate free radicals. The MIR dual-comb laser beam was passed through the Herriott multipass flow reactor and then detected by a photovoltaic HgCdTe (MCT) detector. Afterwards, dual-comb interferograms were recorded and digitized with a 12-bit data-acquisition (DAQ) board at a sampling rate of 500 MS s^−1^. To monitor the time-dependent spectral evolution upon the irradiation of the flowing mixtures, both excimer laser and DAQ board were synchronized with the dual-comb interference signal. In addition, the external trigger of the excimer laser was set to have a time delay by using a pulse delay generator. Additional details of the experimental setup are shown in Methods.

To perform high-resolution time-resolved spectroscopy based on comb-mode-resolved dual-comb technique, we first continuously recorded dual-comb interference signal over tens of milliseconds. The time-domain dual-comb signal was cut every few interferograms and separately Fourier-transformed to generate time-dependent comb-mode-resolved spectra. In the comb-mode-resolved dual-comb technique, each comb line provides one spectral sampling point. The detailed descriptions are provided in Supplementary Note 1 and Supplementary Fig. 1. After Fourier transformation of the time-dependent dual-comb interferograms and data processing with programs, a precursor transmission spectrum before photolysis and time-resolved signal transmission spectra after photolysis can be obtained. Typically, the sample point spacing corresponds to the comb mode spacing, and can be decreased by interleaving. The temporal resolution can be adjusted according to the number of interferograms used to generate each signal spectrum.

### Simultaneous determination of multiple reaction species near 3 μm

**Fig. 2:**
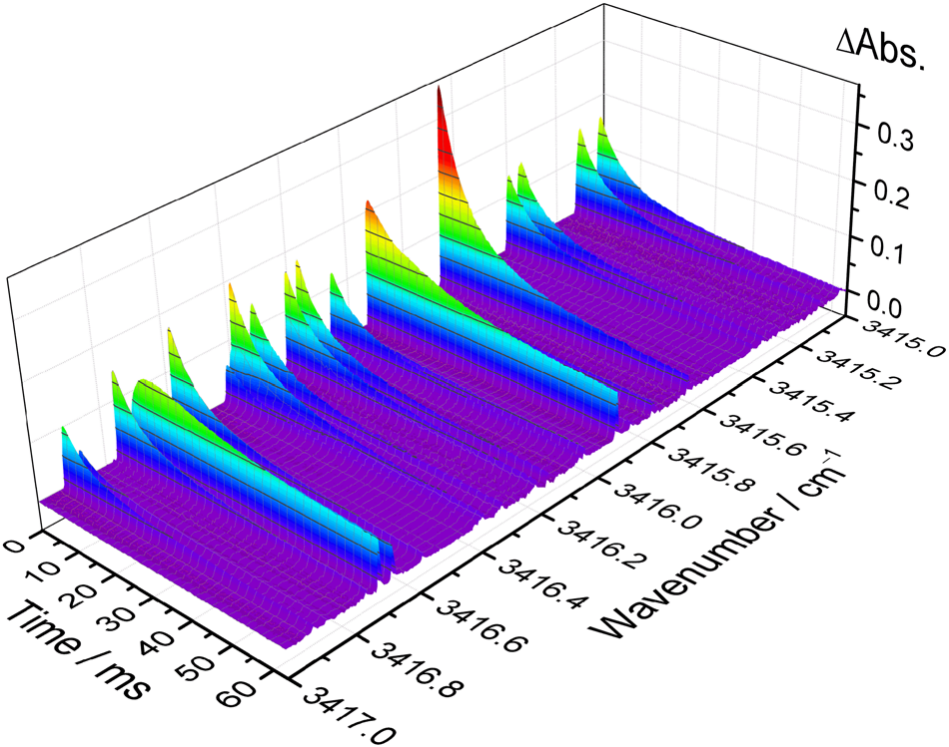
Representative time-resolved dual-comb spectra. The spectra were obtained after the 248-nm irradiation of a flowing mixture of (COCl)_2_/CH_3_OH/O_2_ (1/1.5/20.6, 4.53 Torr, 296 K). The sample point spacing is 146 MHz (~4.87 × 10^−3^ cm^−1^) and the temporal resolution is 400 μs.

To demonstrate high-resolution time-resolved dual-comb spectroscopy, we measured the time-dependent difference absorbance spectra upon the flash photolysis of a flowing mixture of (COCl)_2_/CH_3_OH/O_2_ (1/1.5/20.6) at 4.53 Torr and 296 K, as shown in Fig. 2. The dual-comb spectrometer was set with a comb mode spacing (f_rep_) of 146 MHz (~4.87 × 10^−3^ cm^−1^) and a different repetition frequency (δf) of 0.05 MHz. The time-dependent difference absorbance spectra were derived using the following equation: −ln[T_n_(ν) / T_0_(ν)], where T_n_(ν) represents the signal transmission spectra taken after photolysis and T_0_(ν) represents the precursor transmission spectrum taken before photolysis. Each time-dependent spectrum was obtained through the Fourier transformation of 20 interferograms and averaged over 1000 excimer laser shots. The temporal resolution of the time-resolved spectra was estimated to be 400 μs. To perform transient absorption measurements for the HO_2_ radicals, oxalyl chloride (COCl)_2_ was used as the precursor to efficiently generate Cl atoms upon irradiation with the excimer laser at 248 nm. The Cl atoms reacted with methanol (CH_3_OH) through hydrogen atom abstraction to form HCl and CH_2_OH radicals. Subsequently, CH_2_OH mainly reacted with excessive O_2_ in the system to produce formaldehyde (H_2_CO) and hydroperoxyl radical (HO_2_). The difference absorbance signals from CH_3_OH, H_2_CO, and HO_2_ radicals were simultaneously obtained and distinguished by their distinct temporal profiles (Supplementary Fig. 2).

**Fig. 3:**
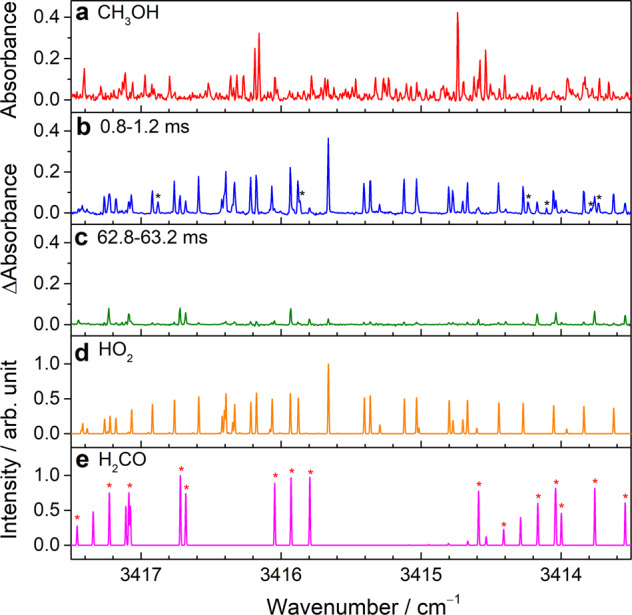
Comparison of the observed and predicted spectra of HO_2_ and H_2_CO. **a** Absorbance spectrum of a flowing mixture of (COCl)_2_/CH_3_OH/O_2_ (1/1.5/20.6, 4.53 Torr, 296 K) before photolysis (−0.4–0 ms). Difference absorbance spectra recorded with a sample point spacing of 146 MHz (~4.87 × 10^−3^ cm^−1^) for **b** 0.8–1.2 and **c** 62.8–63.2 ms after the irradiation of the flowing mixture. The additional lines indicated with a black asterisk in (**b**) are tentatively assigned to the hot band transitions of HO_2_. **d** Simulated spectrum of HO_2_ taken from the HITRAN database^27,29^. **e** Spectrum of the 2ν_2_ band of H_2_CO simulated according to the vibrational-rotational parameters^28^ by using the PGOPHER program^30^. The lines indicated with a red asterisk in **e** represent that they were assigned in the experiment^28^.

Figure 3 shows a comparison of the measured spectra and predicted transition lines of HO_2_ and H_2_CO in the region 3413.5−3417.5 cm^−1^. Figure 3a depicts the absorbance spectrum of the precursor obtained before flash photolysis. At an early period after flash photolysis of the flowing mixture, several absorption lines from the stable product (H_2_CO) and transient HO_2_ radicals were observed, as illustrated in Fig. 3b. Most of the absorption lines in Fig. 3b can be assigned to the transitions of the ν_1_ band of HO_2_^27^ and the 2ν_2_ band of H_2_CO^28^. A simulated spectrum of HO_2_ taken from the HITRAN database^29^ is depicted in Fig. 3d, and a spectrum of H_2_CO generated by the PGOPHER program^30^ with the vibrational-rotational parameters of the 2ν_2_ band of H_2_CO^28^ is presented in Fig. 3e. The absorbance intensities of the HO_2_ radicals reduced mainly due to the self-reaction of HO_2_ radicals with a rate coefficient of 1.7 × 10^‒12^ cm^3^ molecule^‒1^ s^‒1^ at 296 K^31^. These HO_2_ lines exhibited considerably weak absorption signals after tens of milliseconds, as displayed in Fig. 3c. In addition, several additional absorption lines (indicated with a black asterisk in Fig. 3b) were found with the similar temporal profiles as that of the HO_2_ ν_1_ transitions, as shown in Supplementary Fig. 3. Although these additional lines have never been observed under a discharge system in previous studies, we tentatively assigned them to the hot band transitions of HO_2_ according to anharmonic frequency calculations (B3LYP/aug-cc-pVTZ). The calculated center frequencies of the HO_2_ vibrational bands are listed in Supplementary Table 1. The center frequencies of the (110)-(010) and (101)-(001) hot bands of HO_2_ were calculated with red shifts of 16.7 and 3.8 cm^−1^, respectively, from the ν_1_ fundamental band. Each vibrational band may include hundreds of ro-vibrational transitions. To fully assign these additional absorption lines and obtain the ro-vibrational parameters of each band, spectral acquisition would be conducted over the spectral range from 3350 to 3500 cm^**−**1^ in future work.

**Fig. 4:**
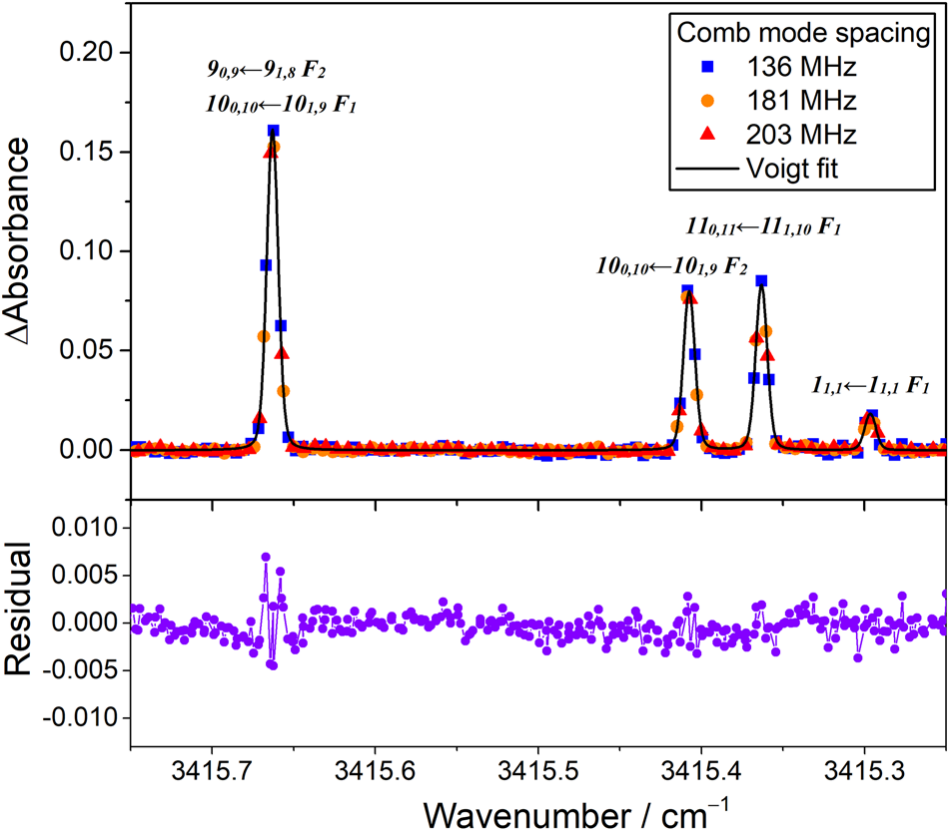
Comparison of measured HO_2_ absorption lines with fitted Voigt profiles. The spectra were measured using the dual-comb spectrometer with different comb mode spacings at 0–0.1 ms after laser photolysis of a flowing mixture of Cl_2_/CH_3_OH/O_2_ (1/0.9/58, 5.94 Torr, 296 K). The absorption lines were curve-fitted using a multi-peak Voigt function. The bottom part of the figure presents the fitting residuals.

To perform quantitative spectral analysis and estimate the concentration of HO_2_ radicals, we selected Cl_2_ as the precursor to produce Cl atoms upon 351-nm photolysis and studied the time-resolved spectra of the HO_2_ radicals with well-known reaction mechanisms. Figure 4 illustrates the difference absorbance spectra of the HO_2_ radicals obtained at 0–0.1 ms after the laser photolysis of a flowing mixture of Cl_2_/CH_3_OH/O_2_ (1/0.9/58, 5.94 Torr, 296 K). To increase the spectral sampling points, we recorded the time-resolved dual-comb spectra by using different comb mode spacings. The interleaved spectrum of the HO_2_ radicals was curve-fitted using a multi-peak Voigt function with a fixed Gaussian width (full width at half maximum [FWHM]) of 220 MHz (which corresponded to Doppler width at 296 K). The Lorentzian width (FWHM) was obtained to be 51 ± 2 MHz, which is comparable to the value of 50 MHz estimated using a broadening coefficient γ_air_ (half width at half maximum [HWHM]) of 0.107 cm^−1^ atm^−1^ ^29^. The line strengths of the HO_2_ transitions were estimated and compared with the values tabulated in the HITRAN database, as listed in Supplementary Table 2. The obtained line strengths were three times higher than those from the HITRAN database^29,32^. In addition, in a recent experiment for line strength measurements of the HO_2_ ν_3_ band under a flash photolysis system, the obtained line strengths were reported to be approximately three times higher than those from previous studies^33^.

### Kinetic studies of the HO_2_+NO reaction

**Fig. 5:**
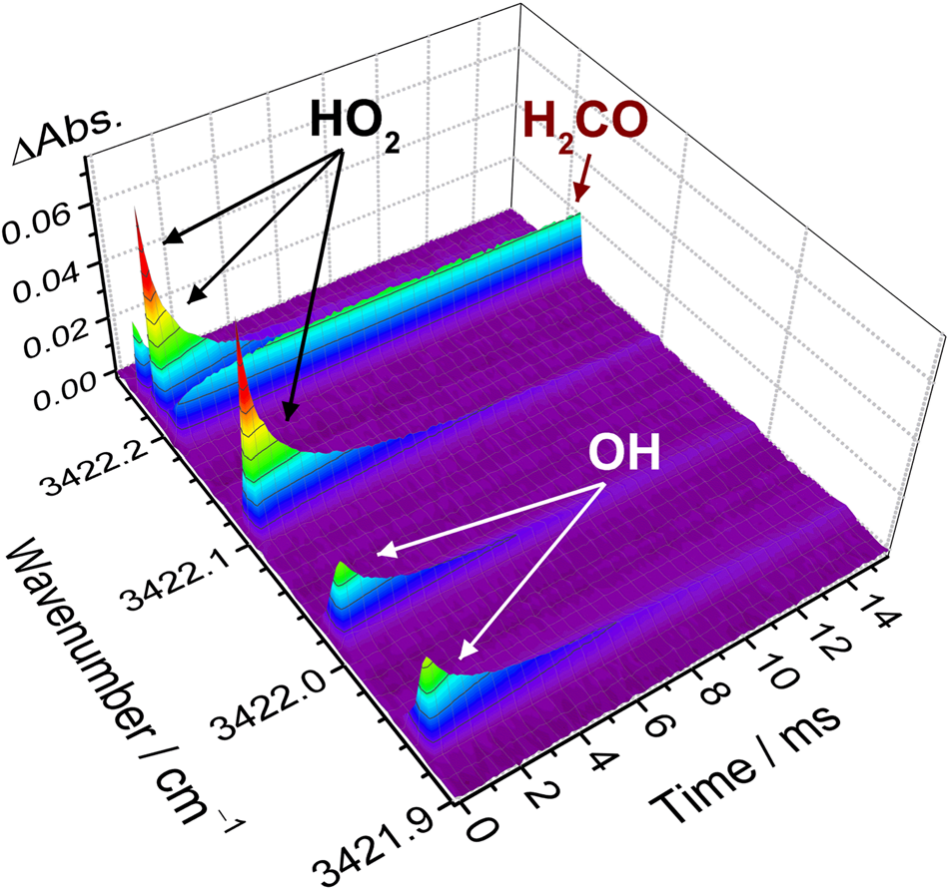
Time-resolved dual-comb spectra in the HO_2_ + NO reaction. The spectra were recorded after photolysis of a flowing mixture of Cl_2_/CH_3_OH/O_2_/NO (1/0.14/10.02/0.017, 6.02 Torr, 296 K) at 351 nm. The sample point spacing is 181 MHz (~6.03 × 10^−3^ cm^−1^) and the temporal resolution is 125 μs.

To investigate the kinetics of the HO_2_ + NO reaction, we first recorded the time-resolved dual-comb spectra upon irradiation of a flowing mixture of Cl_2_/CH_3_OH/O_2_/NO (1/0.14/10.02/0.017, 6.02 Torr, 296 K) at 351 nm, as shown in Fig. 5. The dual-comb spectrometer was set with a comb mode spacing of 181 MHz (~6.03 × 10^−3^ cm^−1^) and a different repetition frequency of 0.08 MHz. Each time-dependent spectrum was generated by Fourier transformation of 10 dual-comb interferograms and averaged over 1600 excimer laser shots. By recording time-resolved spectra at around 3422 cm^−1^, both HO_2_ and OH radicals were simultaneously detected, which is useful for kinetic studies under various experimental conditions.

**Fig. 6:**
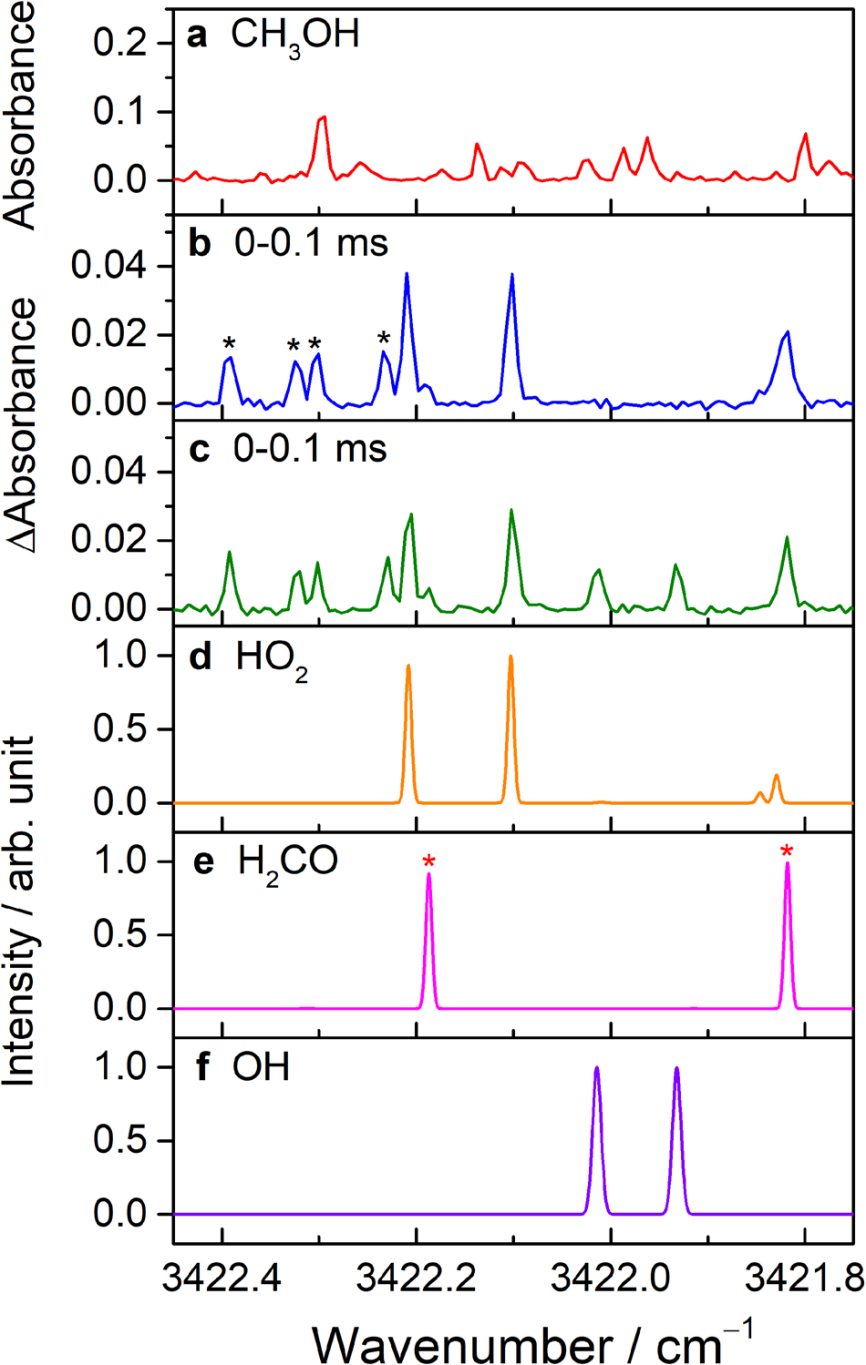
Comparison of the observed and predicted spectra of HO_2_, H_2_CO, and OH. **a** Absorbance spectrum of a flowing mixture of Cl_2_/CH_3_OH/O_2_ (1/0.36/24.5) at 8.24 Torr before photolysis and **b** difference absorbance spectrum recorded at 0–0.1 ms after photolysis. The HO_2_ hot band lines in (**b**) are indicated with a black asterisk. **c** Difference absorbance spectrum recorded at 0–0.1 ms after irradiation of the flowing mixture with [NO]_0_ = 1.72 × 10^14^ molecules cm^−3^. Here, the spectra were measured with a sample point spacing of 181 MHz (~6.03 × 10^−3^ cm^−1^). **d** Simulated spectrum of HO_2_ taken from the HITRAN database^27,29^. **e** Spectrum of the 2ν_2_ band of H_2_CO simulated according to the vibrational-rotational parameters^28^ by using the PGOPHER program^30^. The lines indicated with a red asterisk in **e** represent that they were assigned in the experiment^28^. **f** Simulated spectrum of OH obtained from the HITRAN database^29^.

A comparison of the observed spectra and predicted transition lines of HO_2_, H_2_CO, and OH in the region 3421.75−3422.45 cm^**−**1^ is displayed Fig. 6. An absorbance spectrum recorded before photolysis of a flowing mixture of Cl_2_/CH_3_OH/O_2_ (1/0.36/24.5, 8.24 Torr, 296 K) and a difference absorbance spectrum recorded at 0–0.1 ms after photolysis are depicted in Fig. 6a, b, respectively. In this spectral region, several additional lines indicated with a black asterisk in Fig. 6b were observed and tentatively assigned to be HO_2_ hot band lines according to comparison of the temporal profiles (Supplementary Fig. 4). With the addition of NO in the flow cell, two lines belonging to the absorption of OH radicals were clearly observed without interference with other reaction products, as shown in Fig. 6c. For the kinetic studies, to avoid possible interferences with the H_2_CO and HO_2_ hot band absorption lines, a HO_2_ line at 3422.102 cm^‒1^ and two OH lines at 3421.932 and 3422.014 cm^‒1^ were selected for the analysis of temporal absorbance profiles at different experimental conditions.

**Fig. 7:**
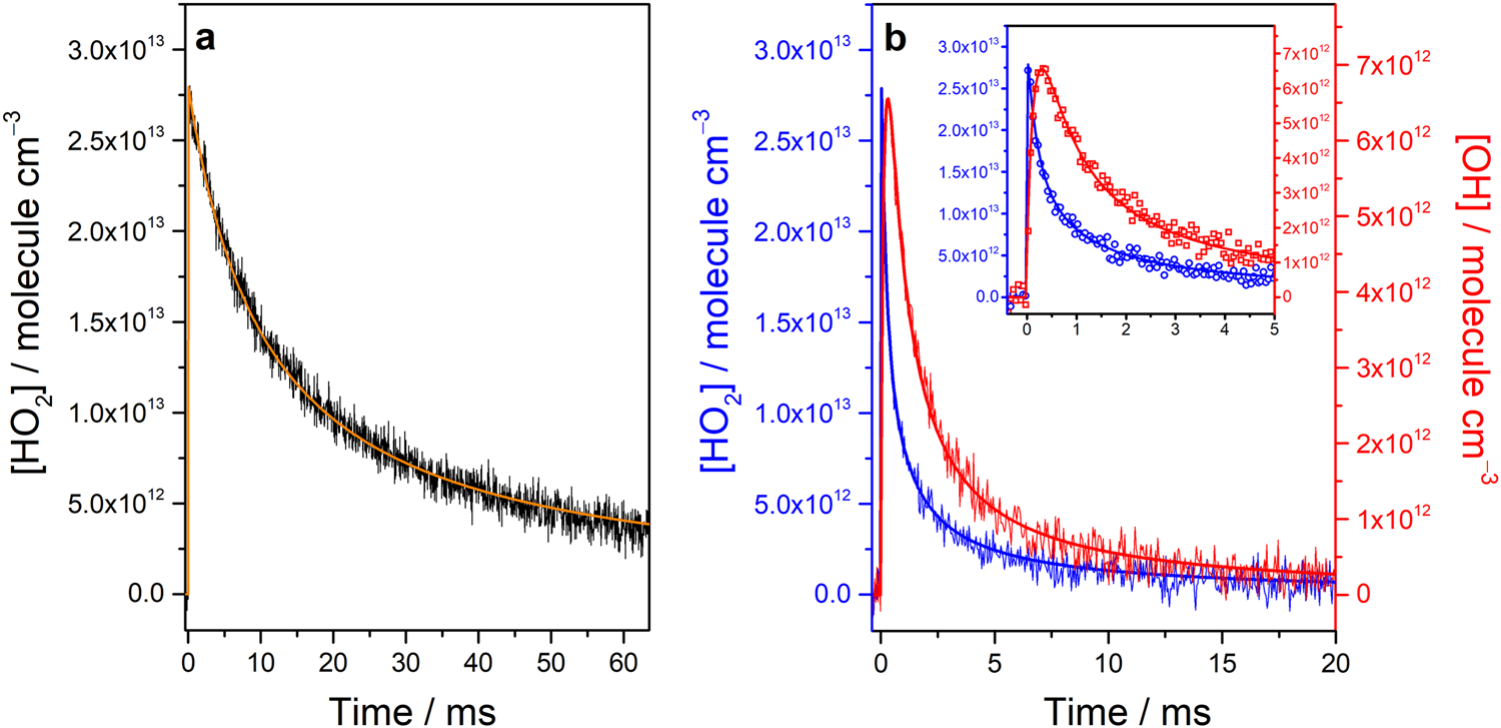
Temporal profiles of the concentrations of HO_2_ and OH radicals. **a** A time trace of the HO_2_ line at 3422.102 cm^‒1^ (black) was recorded with a temporal resolution of 50 μs upon the laser photolysis of a flowing mixture of Cl_2_/CH_3_OH/O_2_/N_2_ (1/0.45/25.7/7.18, 8.33 Torr, 296 K) at 351 nm. **b** Time traces of the HO_2_ line at 3422.102 cm^‒1^ (blue) and the OH line at 3421.932 cm^‒1^ (red) were measured with a temporal resolution of 50 μs upon the irradiation of the flowing mixture with [NO]_0_ = 3.06 × 10^14^ molecule cm^−3^. The thick solid lines represent profiles fitted using the kinetic model. The insert in **b** depicts the temporal profiles from −0.4 to 5 ms.

In kinetic measurements, we first analyzed the time-resolved dual-comb spectrum without the addition of NO to the reaction system and determined the initial concentration of Cl atoms through the model-fitting of the absorbance time trace of HO_2_ lines. With the addition of NO in reaction system, the rate coefficients of the HO_2_ + NO reaction were obtained by analyzing the temporal absorbance profiles of HO_2_ and OH radicals with the kinetic model (Supplementary Table 3). Figure 7a shows a representative time trace of the HO_2_ line at 3422.102 cm^‒1^ taken from the time-resolved spectra, which were measured upon photolysis of a flowing mixture of Cl_2_/CH_3_OH/O_2_/N_2_ (1/0.45/25.7/7.18, 8.33 Torr, 296 K) and obtained with a sample point spacing of 203 MHz (~6.77 × 10^−3^ cm^−1^) and a temporal resolution of 50 μs. The signal to noise ratio (SNR) of this time trace was estimated to be approximately 50 over 1600 photolysis laser shots. The yellow solid line represents the curve that was fitted using the kinetic model. The initial concentration of Cl atoms was determined to be 2.81 × 10^13^ molecules cm^−3^. When [NO]_0_ = 3.06 × 10^14^ molecules cm^−3^ in the reaction system, the temporal absorbance profiles of the HO_2_ and OH lines (indicated with blue and red lines, respectively, in Fig. 7b) were recorded simultaneously and analyzed for determining the first-order rate coefficients for the reaction of HO_2_ with NO. Furthermore, the line strengths of the OH transitions were estimated by analyzing the spectral profiles (Supplementary Fig. 6). The obtained line strength of the OH transitions (S = (1.9 ± 0.6) × 10^‒20^ cm molecule^‒1^) is comparable to the value tabulated in the HITRAN database (S = (2.6 ± 0.5) × 10^‒20^ cm molecule^‒1^)^29,34^.

**Fig. 8:**
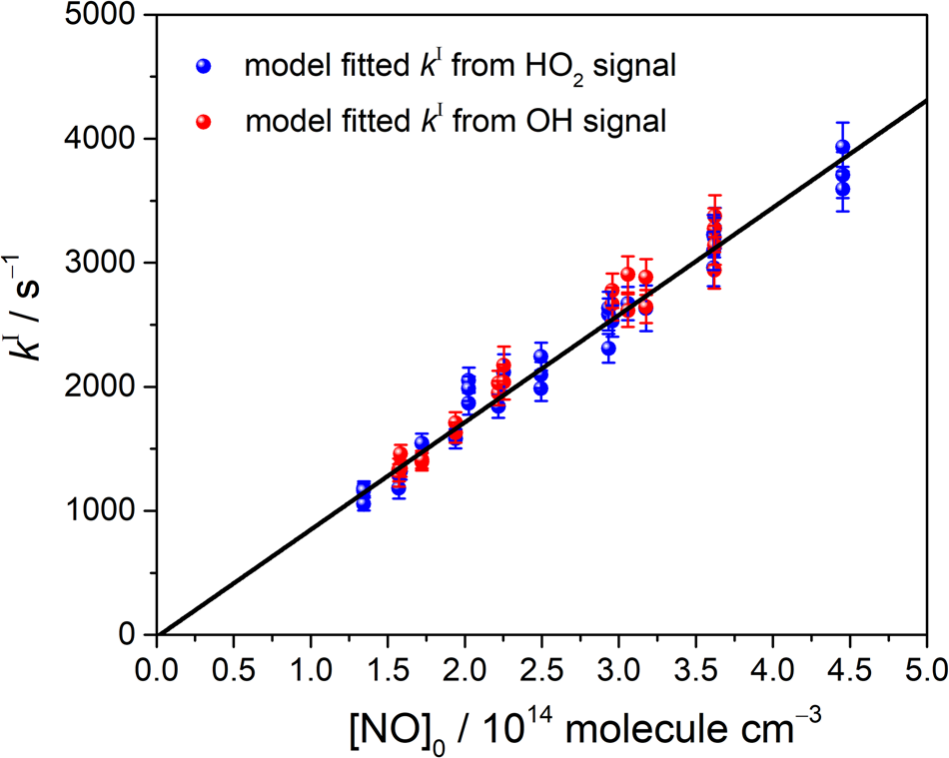
Dependence of the first-order rate coefficient *k*^*I*^ of HO_2_ + NO on [NO]_0_. The error bar represents the fitting error of each *k*^*I*^ obtained by model fitting of the temporal absorbance profiles of HO_2_ and OH radicals. The black line indicates a linear fitting curve with a slope of (8.7 ± 0.3) × 10^‒12^ cm^3^ molecule^‒1^ s^‒1^.

We conducted 17 measurements under total pressures of approximately 8.5 and 17 Torr with [Cl_2_] = (1.7 − 10.4) × 10^15^ molecules cm^−3^, [CH_3_OH] = (2.9 − 3.8) × 10^15^ molecules cm^−3^, [O_2_] = (1.7 − 2.5) × 10^17^ molecules cm^−3^, [Cl]_0_ = (1.1 − 4.3) × 10^13^ molecules cm^−3^, and [NO]_0_ = (1.3 − 4.5) × 10^14^ molecules cm^−3^ at 296 K. Several lines of HO_2_ and OH radicals in the measured time-resolved spectra were selected and analyzed through kinetic model fitting. The bimolecular rate constant of the HO_2_ + NO reaction was derived from the fitted slope of the dependence of the first-order rate coefficient (*k*^I^) on [NO]_0_, as shown in Fig. 8. The details of each experimental condition and the fitted *k*^I^ values are listed in Supplementary Table 4. Considering the slope fitting error (3.4 %) and some errors in the measurements of the flow rates (3 %), temperature (1 %), and pressure (1 %), we estimated the overall standard error to be ~5%. Therefore, the rate coefficient for HO_2_ + NO was determined to be (8.7 ± 0.4) × 10^‒12^ cm^3^ molecule^‒1^ s^‒1^. This result is in agreement with those of other experimental reports^25,35,36^, thus, the adopted method using time-resolved dual-comb spectroscopy is feasible for kinetic studies.

## Discussion

We simultaneously demonstrated multispecies determination under gas-phase chemical reaction systems and studied the kinetics of the HO_2_ + NO reaction by employing time-resolved dual-comb spectroscopy near 3 μm. Quantitative spectral and temporal analyses of the recorded time-resolved dual-comb spectra were performed. The advantage of utilizing the adopted approach for kinetic studies is that we can first record time-dependent dual-comb interferograms and then analyze the Fourier-transformed spectra with different temporal resolutions. A comparison of the temporal profiles of a HO_2_ line at 3415.663 cm^−1^ with temporal resolutions of 25 and 400 μs is displayed in Supplementary Fig. 7a. The SNRs of the time traces with temporal resolutions of 25 and 400 μs were estimated to be 240 and 929, respectively, over 4000 excimer laser shots. Supplementary Fig. 7b shows an evolution of the SNR of the temporal profiles with a temporal resolution of 25 μs as a function of the average number of spectra. The detection limit for the HO_2_ absorption line at 3415.663 cm^−1^ was estimated to be 2.5 × 10^11^ molecules cm^−3^ (with a temporal resolution of 25 μs and average of 4000 shots). Although the sensitivity of current system for HO_2_ detection is lower than that attained in experiments using cavity ring-down spectroscopy in the near infrared region^37,38^, the sensitivity of the adopted method can be improved by coupling the dual-comb laser with a cavity-enhanced reaction cell.

By employing the dual-comb spectrometer in the spectral region of the fundamental OH stretch, we simultaneously determined HO_2_ and OH radicals as well as CH_3_OH molecules. Thus, our adopted method may be suitable for studying the reaction between hydroxyl and methylperoxy radicals^39–41^ and directly obtaining the yield of methanol from the OH + CH_3_O_2_ reaction. For a rough estimation, both methanol and OH radicals may be detected with a detection limit of approximately 1 × 10^11^ molecules cm^−3^ by recording time-resolved spectra at around 3650 cm^−1^.

Moreover, our electro-optic dual-comb spectrometer can be designed with an all-fiber compact system without any complicated phase-lock electronics. Thus, the proposed method is promising for in situ time-resolved measurements and remote sensing to explore the issues in atmospheric chemistry and other applied sciences. Future studies can perform quantitative spectral analysis with rotationally resolved spectra of key radicals, such as organic peroxy radicals (RO_2_) with torsion-CH stretch coupled infrared spectral features^42^. Unstable molecules, such as the simplest Criegee intermediate^2^, which has relatively weak C-H vibrational bands, can also be observed with our time-resolved dual-comb spectrometer coupled with a cavity-enhanced cell.

## Methods

### MIR time-resolved dual-comb spectroscopy

Our MIR electro-optic dual-comb spectrometer is setup using difference frequency generation between a 30 ps electro-optic dual-comb laser at 1050 nm and a widely tunable cw laser in the region 765 − 800 nm^26^. The spectral tunable range of the MIR dual-comb laser is from 2976 to 3548 cm^−1^, and the spectral tunable range can be extended by simply changing the cw lasers. The MIR dual-comb source has a spectral span of 1.8 cm^−1^ and an average power of up to a few mW. The sample point spacing can be adjusted by changing the comb mode spacing, which can be simply tuned from 80 MHz to 4 GHz by adjusting the frequency of the signal generators. All of signal generators and the DAQ board are referenced to a GPS disciplined rubidium clock.

To perform time-resolved dual-comb spectroscopy, the photolysis laser and the DAQ board are synchronized with a multi-channel pulse delay generator, which is triggered by the obtained dual-comb multi-heterodyne signals. Time-domain dual-comb interferograms can thus be continuously recorded before and after excimer laser irradiation. The total acquisition time is 64 ms at a sampling rate of 500 MS s^−1^. The temporal resolution of the adopted method is adjustable and depends on the interferogram length used to generate each signal spectrum. The minimum acquisition time for the generation of a comb-mode-resolved spectrum is the inverse of the different repetition frequency (δf) in the dual-comb system. Therefore, the dual-comb spectrometer can be used to study fast reactions or detect intermediates with a temporal resolution of <1 μs and a sample point spacing >0.008 cm^−1^ ^26^. In the experiments, each time-dependent spectrum is obtained through Fourier transformation of at least five interferograms. The detailed description of data processing is presented in Supplementary Note 1. For absolute frequency calibration, the time-resolved spectra of the reaction cell and the dual-comb spectra of a N_2_O reference cell are simultaneously measured. The wavenumbers of the recorded dual-comb spectra are calibrated based on the simulated spectra of N_2_O molecules obtained from the HITRAN database^29^. Thus, the accuracy of absolute frequency measurements is expected to be <30 MHz (0.001 cm^−1^). More precise calibration can be achieved by measuring frequencies of the cw lasers with a metrology comb system^10^.

### Herriott multipass reaction cell

The Herriott cell consists of a pair of 2-inch concave mirrors with a 25-mm-diameter center hole and 4.75-mm-diameter off-axis hole in each mirror for passage of the ultraviolet (UV) photolysis beam and MIR dual-comb beam, respectively. The MIR dual-comb beam is coupled into the cell and then multi-reflected between the two mirrors separated by a distance of 655 mm. The Herriott cell is designed to allow 63 passes of the MIR beam. A total path length is determined to be (41.8 ± 0.6) m and the length of overlap between the UV and MIR beams is estimated to be (24.5 ± 5.9) m (Supplementary Note 2 and Supplementary Fig. 5). To generate HO_2_ radicals and study the HO_2_ + NO reaction, we performed flash photolysis of flowing mixtures of (COCl)_2_ or Cl_2_/CH_3_OH/O_2_/N_2_/NO. The flow rates of each precursor are controlled using calibrated mass flow controllers. A small stream of N_2_ or O_2_ is used to purge the Herriott mirrors. The total flow rates are typically >1300 standard cm^3^ min^−1^ at low total pressure (< 10 Torr). The partial pressures of each precursor in the Herriot cell are calculated according to the ratios of the flow rates and the total pressure.

## Supplementary Information

Supplementary Information

## Data Availability

The data supporting the findings of this study are available within the article and its Supplementary Information and from the corresponding author upon reasonable request.

## References

[1] Manning CJ, Palmer RA, Chao JL (1991). Step-scan Fourier-transform infrared spectrometer. Rev. Sci. Instrum..

[2] Su Y-T, Huang Y-H, Witek HA, Lee Y-P (2013). Infrared absorption spectrum of the simplest Criegee intermediate CH_2_OO. Science.

[3] Lin H-Y (2015). Infrared identification of the Criegee intermediates *syn-* and *anti-*CH_3_CHOO, and their distinct conformation-dependent reactivity. Nat. Commun..

[4] Hui AO, Fradet M, Okumura M, Sander SP (2019). Temperature dependence study of the kinetics and product yields of the HO_2_ + CH_3_C(O)O_2_ reaction by direct detection of OH and HO_2_ radicals using 2f-IR wavelength modulation spectroscopy. J. Phys. Chem. A.

[5] Luo P-L, Endo Y, Lee Y-P (2018). Identification and self-reaction kinetics of Criegee intermediates *syn*-CH_3_CHOO and CH_2_OO via high-resolution infrared spectra with a quantum-cascade laser. J. Phys. Chem. Lett..

[6] Fortier T, Baumann E (2019). 20 years of developments in optical frequency comb technology and applications. Commun. Phys..

[7] Picqué N, Hänsch TW (2019). Frequency comb spectroscopy. Nat. Photon..

[8] Kowligy AS (2019). Infrared electric field sampled frequency comb spectroscopy. Sci. Adv..

[9] Weichman ML (2019). Broadband molecular spectroscopy with optical frequency combs. J. Mol. Spectrosc..

[10] Yan M (2017). Mid-infrared dual-comb spectroscopy with electro-optic modulators. Light Sci. Appl..

[11] Parriaux A, Hammani K, Millot G (2020). Electro-optic frequency combs. Adv. Opt. Photon..

[12] Fleisher AJ (2014). Mid-infrared time-resolved frequency comb spectroscopy of transient free radicals. J. Phys. Chem. Lett..

[13] Abbas MA (2019). Time-resolved mid-infrared dual-comb spectroscopy. Sci. Rep..

[14] Bergevin J (2018). Dual-comb spectroscopy of laser-induced plasmas. Nat. Commun..

[15] Zhang Y (2019). Time-resolved dual-comb measurement of number density and temperature in a laser-induced plasma. Opt. Lett..

[16] Draper AD (2019). Broadband dual-frequency comb spectroscopy in a rapid compression machine. Opt. Express.

[17] Bjork BJ (2016). Direct frequency comb measurement of OD + CO → DOCO kinetics. Science.

[18] Bui TQ (2018). Direct measurements of DOCO isomers in the kinetics of OD + CO. Sci. Adv..

[19] Pinkowski NH (2020). Dual-comb spectroscopy for high-temperature reaction kinetics. Meas. Sci. Technol..

[20] Klocke JL (2018). Single-shot sub-microsecond mid-infrared spectroscopy on protein reactions with quantum cascade laser frequency combs. Anal. Chem..

[21] Kim J, Cho B, Yoon TH, Cho M (2018). Dual-frequency comb transient absorption: broad dynamic range measurement of femtosecond to nanosecond relaxation processes. J. Phys. Chem. Lett..

[22] Stone D, Whalley LK, Heard DE (2012). Tropospheric OH and HO_2_ radicals: field measurements and model comparisons. Chem. Soc. Rev..

[23] Donahue NM (2011). The reaction that wouldn’t quit. Nat. Chem..

[24] Wennberg PO (1994). Removal of stratospheric O_3_ by radicals: In situ measurements of OH, HO_2_, NO, NO_2_, ClO, and BrO. Science.

[25] Bardwell MW (2003). Kinetics of the HO_2_+ NO reaction: a temperature and pressure dependence study using chemical ionisation mass spectrometry. Phys. Chem. Chem. Phys..

[26] Luo P-L, Horng E-C, Guan Y-C (2019). Fast molecular fingerprinting with a coherent, rapidly tunable dual-comb spectrometer near 3 μm. Phys. Chem. Chem. Phys..

[27] Yamada C, Endo Y, Hirota E (1983). Difference frequency laser spectroscopy of the ν_1_ band of the HO_2_ radical. J. Chem. Phys..

[28] Tan TL, A’dawiah R, Ng LL (2017). The 2ν_2_ bands of H_2_^12^CO and H_2_^13^CO by high-resolution FTIR spectroscopy. J. Mol. Spectrosc..

[29] Hitran database. http://hitran.org/.

[30] Western, C. M. PGOPHER, a program for simulating rotational structure, http://pgopher.chm.bris.ac.uk/.

[31] Tang Y, Tyndall GS, Orlando JJ (2010). Spectroscopic and kinetic properties of HO_2_ radicals and the enhancement of the HO_2_ self reaction by CH_3_OH and H_2_O. J. Phys. Chem. A.

[32] Zahniser MS, McCurdy KE, Stanton AC (1989). Quantitative spectroscopic studies of the hydroperoxo radical: band strength measurements for the ν_1_ and ν_2_ vibrational bands. J. Phys. Chem..

[33] Sakamoto Y, Tonokura K (2012). Measurements of the absorption line strength of hydroperoxyl radical in the ν_3_ band using a continuous wave quantum cascade laser. J. Phys. Chem. A.

[34] Goldman A (1998). Updated line parameters for OH X^2^II−X^2^II (ν",ν′) transitions. J. Quan. Spec. Rad. Trans..

[35] Jemi-Alade AA, Thrush BA (1990). Reactions of HO_2_ with NO and NO_2_ studied by mid-infrared laser magnetic resonance. J. Chem. Soc. Faraday Trans..

[36] Howard CJ (1979). Temperature dependence of the reaction HO_2_+NO→OH+NO_2_. J. Chem. Phys..

[37] Gianella M (2019). Sensitive detection of HO_2_ radicals produced in an atmospheric pressure plasma using Faraday rotation cavity ring-down spectroscopy. J. Chem. Phys..

[38] Assaf E, Fittschen C (2016). Cross section of OH radical overtone transition near 7028 cm^–1^ and measurement of the rate constant of the reaction of OH with HO_2_ radicals. J. Phys. Chem. A.

[39] Assaf E (2017). The reaction between CH_3_O_2_ and OH radicals: Product yields and atmospheric implications. Environ. Sci. Technol..

[40] Müller J-F (2016). The reaction of methyl peroxy and hydroxyl radicals as a major source of atmospheric methanol. Nat. Commun..

[41] Caravan RL (2018). The reaction of hydroxyl and methylperoxy radicals is not a major source of atmospheric methanol. Nat. Commun..

[42] Huang M (2017). Modeling the CH stretch/torsion/rotation couplings in methyl peroxy (CH_3_OO). J. Phys. Chem. A.

