# Supplementary material for: Simultaneous determination of transient free radicals and reaction kinetics by high-resolution time-resolved dual-comb spectroscopy
Source: Commun Chem. 2020 Jul 30;3:95. doi: 10.1038/s42004-020-00353-6 (PMC9814257; doi:10.1038/s42004-020-00353-6)
Supplement: Supplementary file 1 — Supplementary Information [file 42004_2020_353_MOESM1_ESM.pdf]

Supplementary Information for "Simultaneous Determination of Transient Free Radicals and Reaction Kinetics by High-Resolution Time-Resolved Dual-Comb Spectroscopy"

Pei-Ling Luo[1]* & Er-Chien Horng[1]

[1]Institute of Atomic and Molecular Sciences, Academia Sinica, Taipei 10617, Taiwan.
*E-mail: plluo@gate.sinica.edu.tw

Supplementary Note 1. Time-resolved spectroscopy based on the comb-mode-resolved dual-comb technique

In time-resolved spectroscopy based on the comb-mode-resolved dual-comb technique, each comb line provides one spectral sampling point. Therefore, the spectral sampling spacing is typically equal to the comb mode spacing. The temporal resolution can be adjusted by changing the length of the dual-comb interferogram used to generate each time-dependent spectrum. Supplementary Fig. 1 displays a comparison of the comb-mode-resolved dual-comb spectra generated through the Fourier transformation of 20 dual-comb interferograms and 100 averages (case 1) as well as five dual-comb interferograms and 400 averages (case 2). Although each comb line has a relatively broad linewidth and low intensity in case 2, the obtained transmittance spectrum in case 2 is typically the same as that obtained in case 1.

To perform time-resolved spectroscopy, dual-comb interference signals are recorded and digitized with a data acquisition board. The time-dependent transmission spectra can be obtained after the Fourier transformation of time-dependent dual-comb interferograms, spectral normalization with a spectrum of the evacuated cell, and simple baseline correction. To analyze the spectral variation after flash photolysis, the difference absorbance spectra are derived using the following formula: $[\mathrm{Abs}]_n - [\mathrm{Abs}]_0 = -\ln[T_n(\nu)\ /\ T_0(\nu)]$, where $[\mathrm{Abs}]_n = -\ln[T_n(\nu)]$ represents the signal absorbance spectra taken after photolysis and $[\mathrm{Abs}]_0 = -\ln[T_0(\nu)]$ represents the precursor absorbance spectrum taken before photolysis.

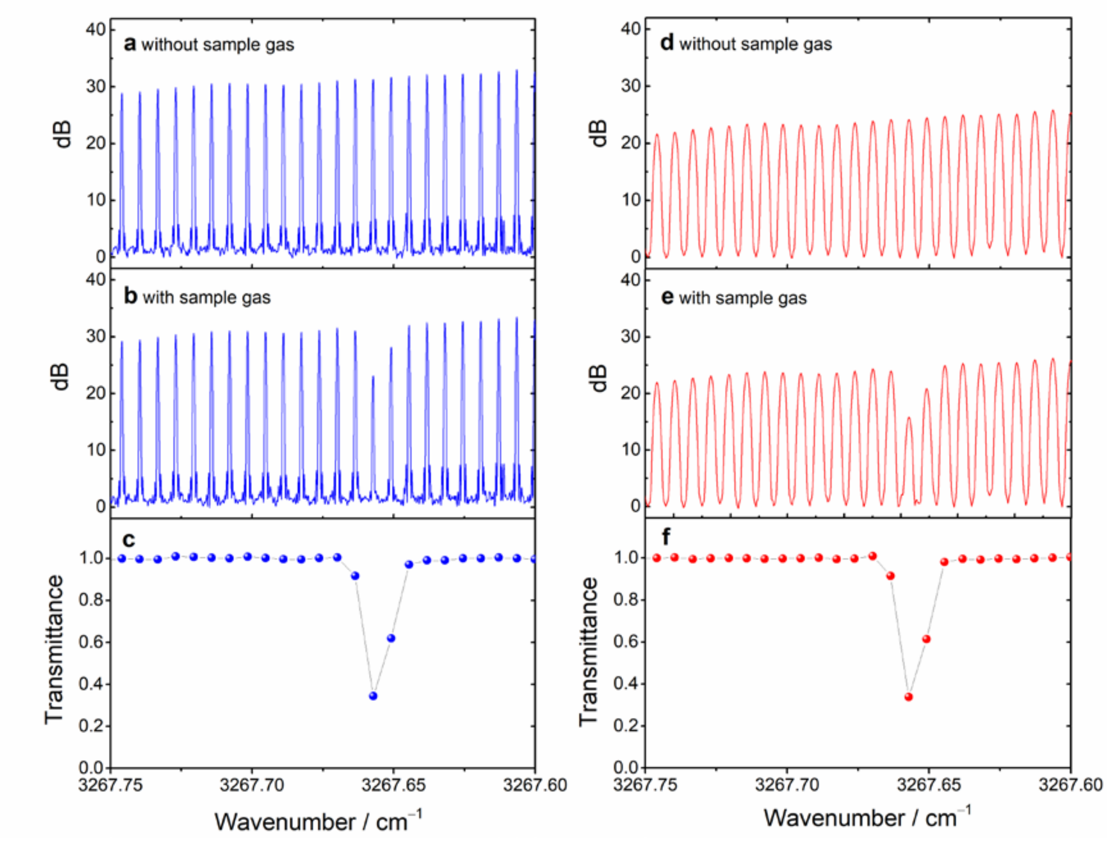

Supplementary Figure 1. Comb-mode-resolved spectra (a) without sample gas and (b) with sample gas obtained through Fourier transformation of 20 dual-comb interferograms and 100 averages. (c) A transmittance spectrum derived by dividing the comb mode peaks in (b) by the comb mode peaks in (a). Comb-mode-resolved spectra (d) without sample gas and (e) with sample gas obtained through Fourier transformation of five dual-comb interferograms and 400 averages. (f) A transmittance spectrum derived by dividing the comb mode peaks in (e) by the comb mode peaks in (f). Here, the dual-comb spectrometer was set with a comb-mode spacing (f_{rep}) of 190 MHz and a δf of 0.07 MHz. The total data recording time was 28.571 ms at a sampling rate of 500 MS s^{-1}. A 14.5-cm-length sample cell was filled with pure C_2H_2 gas at 300 mTorr and 296 K.

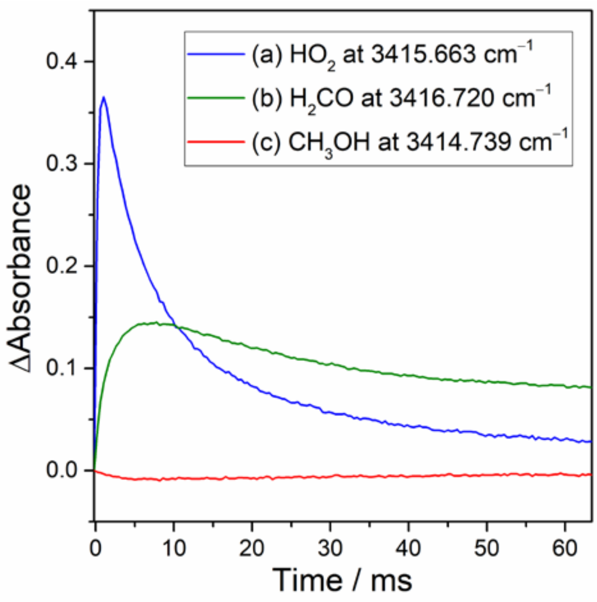

Supplementary Figure 2. Comparison of the temporal profiles of the lines of HO_2, H_2CO, and CH_3OH. The data were taken from the time-resolved spectrum with a spectral sampling spacing of 146 MHz (~4.87×10^{-3} cm^{-1}) and a temporal resolution of 400 μs that was measured upon the irradiation of a flowing mixture of $(COCl)_2/CH_3OH/O_2$ (1/1.5/20.6, 4.53 Torr, 296 K) at 248 nm.

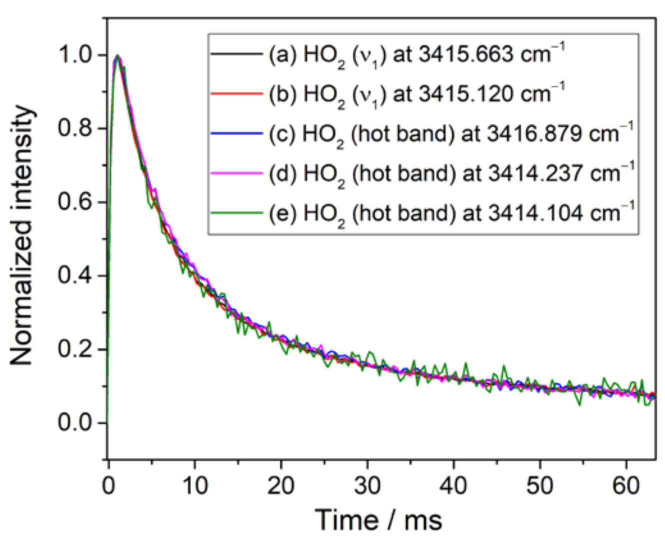

Supplementary Figure 3. Comparison of the temporal profiles of the lines of the ν_1 band of HO_2 and the lines of the HO_2 hot band. The data were taken from the time-resolved spectrum with a spectral sampling spacing of 146 MHz (~4.87×10^{-3} cm^{-1}) and a temporal resolution of 400 μs that was measured upon the irradiation of a flowing mixture of $(COCl)_2/CH_3OH/O_2$ (1/1.5/20.6, 4.53 Torr, 296 K) at 248 nm.

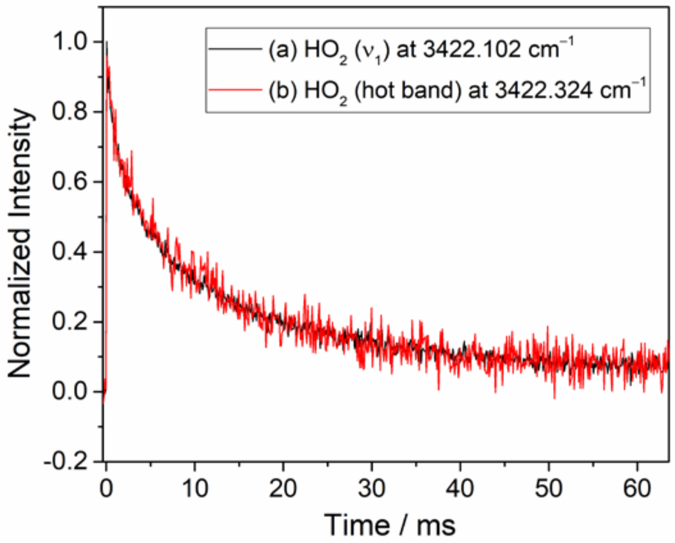

Supplementary Figure 4. Comparison of the temporal profiles of a line of the HO_2 ν_1 band at 3422.102 cm^{-1} and a line of the HO_2 hot band at 3422.324 cm^{-1}. The data were taken from the time-resolved spectrum with a spectral sampling spacing of 181 MHz (~6.03×10^{-3} cm^{-1}) and a temporal resolution of 100 μs that was measured upon irradiation of a flowing mixture of $Cl_2/CH_3OH/O_2$ (1/0.36/24.5, 8.33 Torr, 296 K) at 351 nm.

Supplementary Table 1. Anharmonic frequencies of the HO_2 vibrational bands calculated using the B3LYP/aug-cc-pVTZ method.

Fundamental Bands	Anharmonic calculation / cm^{-1}
(100)-(000)	3387.274
(010)-(000)	1389.672
(001)-(000)	1135.285
Overtones	Anharmonic calculation / cm^{-1}
(200)-(000)	6581.471
(020)-(000)	2753.174
(002)-(000)	2257.557
Combination	Anharmonic calculation / cm^{-1}
(110)-(000)	4760.244
(101)-(000)	4518.759
(011)-(000)	2511.476

,

Supplementary Note 2. Estimation of the overlapping length between the photolysis and MIR beams

The MIR dual-comb beam was coupled into the cell and then multi-reflected between the two mirrors separated by 655 mm and the Herriott cell was designed to allow 63 passes of the MIR beam. The total path length of the MIR beam was determined to be 41.8 m with an uncertainty of ~1.4 %. To estimate the length of overlap between the photolysis and MIR beams, we recorded the absorbance spectrum before photolysis (Supplementary Fig. 5a) and the difference absorbance spectrum at 0–0.1 ms after photolysis (Supplementary Fig. 5b). Before laser photolysis, the absorbance signals of CH_3OH can be obtained to be $[Abs]_{CH3OH} = \sigma n L_{tot}$, where σ is the absorption cross section, n is the concentration, and L_{tot} is the total path length of the MIR beam. After laser photolysis, the difference absorbance signals of CH_3OH can be derived to be $\Delta[Abs]_{CH3OH} = \sigma \Delta n L_{eff}$, where Δn represents the reduced concentration of CH_3OH, which corresponds to the initial concentration of Cl atoms ($[Cl]_0$), and L_{eff} represents the overlapping path of the photolysis and MIR beams. The absorption cross section (σ) of CH_3OH can be estimated through the analysis of the absorbance spectrum taken before photolysis. The $[Cl]_0$ was estimated with a value of 5.93×10^{13} molecules cm^{-3} by model fitting of the temporal profile of a HO_2 line at 3415.120 cm^{-1} and the $\Delta[Abs]_{CH3OH}$ was obtained with a value of $(-1.4\pm0.3)\times10^{-3}$ for a line peak of CH_3OH at 3414.739 cm^{-1} (Supplementary Fig. 5c). Considering the errors of the HO_2 self-reaction rate (10 %)[1], $\Delta[Abs]_{CH3OH}$ (21 %), and L_{tot} (1.4 %) as well as errors in measurements of the flow rates (3 %), temperature (1 %), and pressure (1 %), we estimated the overall uncertainty to be ~24 %. Hence, the overlap path between the photolysis and MIR beams was determined to be (24.5±5.9) m.

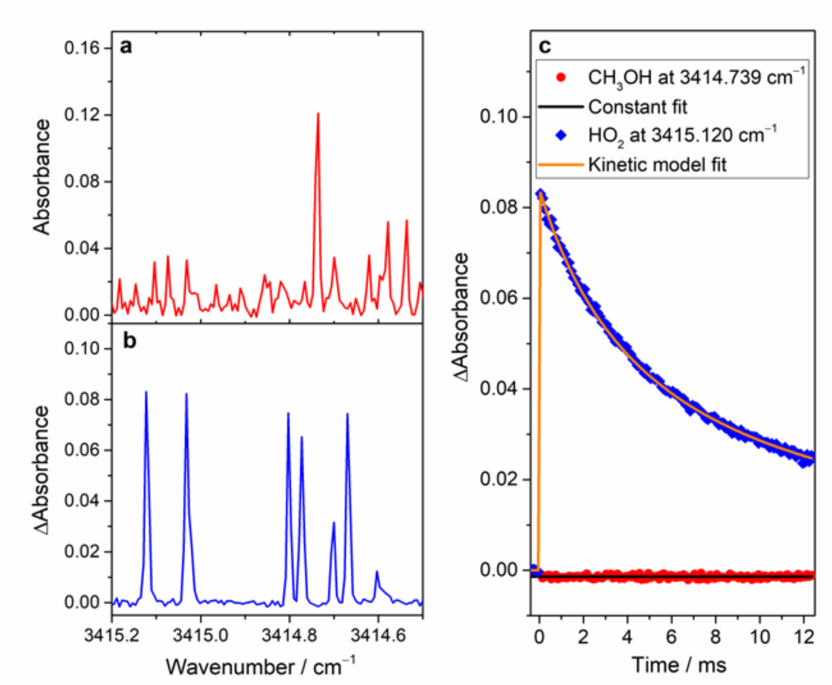

Supplementary Figure 5. (a) Absorbance spectrum of a flowing mixture of $Cl_2/CH_3OH/O_2$ (1/0.91/56, 5.93 Torr, 296 K) before photolysis. (b) Difference absorbance spectrum recorded at 0–0.1 ms after laser photolysis. (c) Temporal profiles of a CH_3OH line at 3414.739 cm^{-1} and a HO_2 line at 3415.120 cm^{-1}. The $[Cl]_0$ was obtained to be 5.93×10^{13} molecules cm^{-3} by model fitting of the time trace of the HO_2 line. The constant fitted value of the time trace of CH_3OH was obtained to be -1.4×10^{-3}, with a standard deviation of 3×10^{-4}. Here, the dual-comb spectrometer was set with a comb mode spacing of 181 MHz (~6.03×10^{-3} cm^{-1}) and a different repetition frequency of 0.1 MHz.

Supplementary Table 2. Comparison of the obtained line strengths of the HO_2 transitions with the line strengths tabulated in the HITRAN database[2].

Transition	Obtained line center[a] / cm^{-1}	Obtained line strength[b,c]	Obtained relative intensity	HITRAN line strength[b,d]	HITRAN relative intensity
$9_{0,9}\leftarrow 9_{1,8}$ F_2<br>$10_{0,10}\leftarrow 10_{1,9}$ F_1	3415.6631	1.08×10^{-20}	1.000	3.45×10^{-21}[e]	1.000
$10_{0,10}\leftarrow 10_{1,9}$ F_2	3415.4076	5.19×10^{-21}	0.481	1.65×10^{-21}	0.478
$11_{0,11}\leftarrow 11_{1,10}$ F_1	3415.3632	5.40×10^{-21}	0.500	1.75×10^{-21}	0.507
$1_{1,1}\leftarrow 1_{1,1}$ F_1	3415.2965	1.19×10^{-21}	0.110	4.10×10^{-22}	0.119

[a] Spectra are calibrated using a N_2O reference cell, and the uncertainty of obtained line center is estimated to be <0.001 cm^{-1}.

[b] Line strength in cm $molecule^{-1}$.

[c] The line strength is derived by A/nL_{eff}, A is the integrated area of the absorbance spectrum, n is the concentration of HO_2 radicals obtained through model-fitting of the absorbance time trace of HO_2 lines ($n = 6.02\times10^{13}$ molecules cm^{-3}), and L_{eff} is the overlapping path of the photolysis and MIR beams (L_{eff} = 24.5 m). The overall uncertainty, including the errors in the integrated absorbance area, concentration of HO_2 radicals, and effective absorption path, is estimated to be 26%.

[d] The HITRAN line strength is calculated on the basis of previous measurements under a flow discharge system[2,3]. The uncertainty of the HITRAN line strength is approximately 30%.

[e] Sum of the line strengths of the $9_{0,9}\leftarrow 9_{1,8}$ F_2 and $10_{0,10}\leftarrow 10_{1,9}$ F_1 transitions.

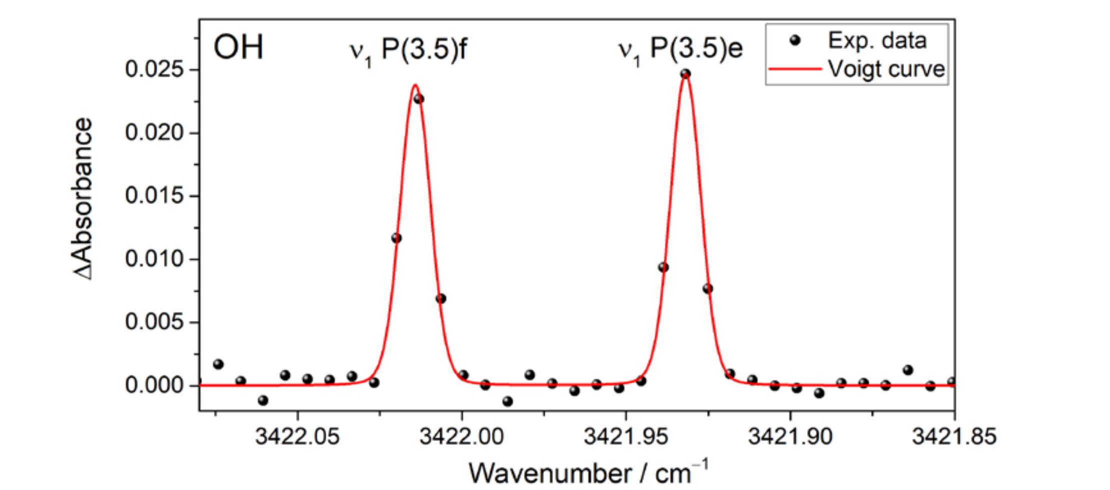

Supplementary Figure 6. Difference absorbance spectrum of the OH radicals with a spectral resolution of 203 MHz (~6.77×10^{-3} cm^{-1}) recorded at 300–350 μs after the laser photolysis of a flowing mixture of $Cl_2/CH_3OH/O_2/N_2/NO$ (1/0.45/25.7/7.18/0.038, 8.33 Torr, 296 K). The estimated [OH] value is 6.57×10^{12} molecules cm^{-3} at 300–350 μs after photolysis. The two absorption lines are fitted using the Voigt function with a fixed Gaussian width (FWHM) of 307 MHz (which corresponds to the Doppler width at 296 K) and a fixed Lorentzian width (FWHM) of 35 MHz (obtained using a broadening coefficients of γ_{air} (HWMH) = 0.053 cm^{-1} atm^{-1})[2]. The estimated line strength of these two transitions is (1.9±0.6) $\times10^{-20}$ cm $molecule^{-1}$, which is comparable to the line strength tabulated in the HITRAN database (S = (2.6±0.5) $\times10^{-20}$ cm $molecule^{-1}$)[2,4].

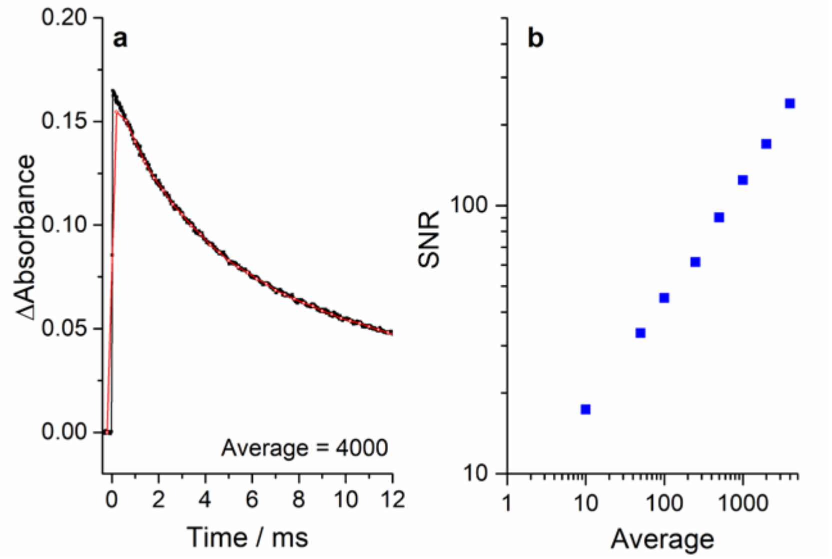

Supplementary Figure 7. (a) Comparison of the temporal profiles of a HO_2 line at 3415.663 cm^{-1} with temporal resolutions of 25 μs (black) and 400 μs (red). The signal-to-noise ratios (SNRs) of the black and red time traces are 240 and 929, respectively. (b) Evolution of the SNR of the temporal profiles of a HO_2 line at 3415.663 cm^{-1} with a temporal resolution of 25 μs as a function of the average number of spectra. Here, the dual-comb spectrometer was set with a comb-mode spacing of 203 MHz (~6.77×10^{-3} cm^{-1}) and a different repetition frequency of 0.2 MHz. The $[HO_2]_0$ was estimated to be 6.11×10^{13} molecules cm^{-3} through model-fitting of the temporal profiles.

Supplementary Table 3. Kinetic model and rate coefficients used in the fitting for the HO_2 + NO reaction.

	Reaction	Rate coefficient [a]	Ref.
k_1	$Cl + CH_3OH \rightarrow CH_2OH + HCl$	5.5×10^{-11}	5
k_2	$CH_2OH + O_2 \rightarrow HO_2 + H_2CO$	9.1×10^{-12}	5
k_3	$CH_2OH + Cl \rightarrow H_2CO + HCl$	6.6×10^{-10}	6
k_4	$CH_2OH + CH_2OH \rightarrow$ products	1.5×10^{-11}	7
k_5	$CH_2OH + Cl_2 \rightarrow ClCH_2OH + Cl$	2.9×10^{-11}	8
k_6	$HO_2 + Cl \rightarrow HCl + O_2$	3.5×10^{-11}	5
k_7	$HO_2 + Cl \rightarrow OH + ClO$	1.0×10^{-11}	5
k_8	$HO_2 + CH_2OH \rightarrow$ products	6.0×10^{-11}	9
k_9	$HO_2 + HO_2 \rightarrow H_2O_2 + O_2$	1.7×10^{-12}	1
k_{10}	$HO_2 + H_2CO \rightarrow HOCH_2OO$	6.3×10^{-14}	10
k_{11}	$H_2CO + Cl \rightarrow HCl + HCO$	7.3×10^{-11}	5
k_{12}	$HCO + O_2 \rightarrow HO_2 + CO$	5.2×10^{-12}	5
k_{13}	$HO_2 + NO \rightarrow OH + NO_2$	$k^{I} = k_{13}\times[NO]_0$, fitted	
k_{14}	$CH_2OH + NO \rightarrow CH_2(OH)NO$	2.5×10^{-11}	11
k_{15}	$OH + HO_2 \rightarrow H_2O + O_2$	1.1×10^{-10}	5
k_{16}	$OH + H_2CO \rightarrow H_2O + HCO$	8.4×10^{-12}	5
k_{17}	$OH + H_2O_2 \rightarrow HO_2 + H_2O$	1.8×10^{-12}	5
k_{18}	$OH + CH_3OH \rightarrow CH_2OH + H_2O$	9.0×10^{-13}	5
k_{19}	$OH + OH \rightarrow H_2O + O$	1.8×10^{-12}	5

[a] Rate coefficient in cm^3 $molecule^{-1}$ s^{-1}.

Supplementary Table 4. Summary of the experimental conditions and fitted first-order rate coefficients

Expt.	Probe line /cm^{-1}	[Cl_2] /$10^{15\ a}$	[CH_3OH] /$10^{15\ a}$	[O_2] /$10^{17\ a}$	$[Cl]_0^b$ /$10^{13\ a}$	$[NO]_0$ /$10^{14\ a}$	P_T /Torr	k^I /s^{-1}
1	3422.102	10.40	3.76	2.51	4.33	1.72	8.24	1544
	3421.932	10.40	3.76	2.51	4.33	1.72	8.24	1395
	3422.014	10.40	3.76	2.51	4.33	1.72	8.24	1412
2	3422.102	10.40	3.76	2.51	4.33	3.18	8.24	2632
	3421.932	10.40	3.76	2.51	4.33	3.18	8.24	2647
	3422.014	10.40	3.76	2.51	4.33	3.18	8.24	2884
3	3415.663	1.71	2.94	1.68	1.06	1.34	17.03 [c]	1179
	3415.408	1.71	2.94	1.68	1.06	1.34	17.03 [c]	1164
	3415.363	1.71	2.94	1.68	1.06	1.34	17.03 [c]	1055
4	3415.663	1.71	2.94	1.68	1.06	2.93	17.03 [c]	2583
	3415.408	1.71	2.94	1.68	1.06	2.93	17.03 [c]	2635
	3415.363	1.71	2.94	1.68	1.06	2.93	17.03 [c]	2310
5	3415.663	1.71	2.94	1.68	1.06	2.03	17.03 [c]	2052
	3415.408	1.71	2.94	1.68	1.06	2.03	17.03 [c]	1868
	3415.363	1.71	2.94	1.68	1.06	2.03	17.03 [c]	1985
6	3415.663	6.07	3.62	2.07	2.60	3.61	8.54 [c]	3225
	3415.408	6.07	3.62	2.07	2.60	3.61	8.54 [c]	3095
	3415.363	6.07	3.62	2.07	2.60	3.61	8.54 [c]	2961
7	3415.663	6.07	3.62	2.07	2.60	4.45	8.54 [c]	3934
	3415.408	6.07	3.62	2.07	2.60	4.45	8.54 [c]	3594
	3415.363	6.07	3.62	2.07	2.60	4.45	8.54 [c]	3707
8	3415.663	6.07	3.62	2.07	2.60	2.50	8.54 [c]	1986
	3415.408	6.07	3.62	2.07	2.60	2.50	8.54 [c]	2244
	3415.363	6.07	3.62	2.07	2.60	2.50	8.54 [c]	2096
9	3422.102	8.06	3.62	2.08	2.81	3.62	8.61 [c]	3203
	3421.932	8.06	3.62	2.08	2.81	3.62	8.61 [c]	2939
	3422.014	8.06	3.62	2.08	2.81	3.62	8.61 [c]	3275
10	3422.102	8.06	3.62	2.08	2.81	3.06	8.61 [c]	2671
	3421.932	8.06	3.62	2.08	2.81	3.06	8.61 [c]	2615
	3422.014	8.06	3.62	2.08	2.81	3.06	8.61 [c]	2907
11	3422.102	8.06	3.62	2.08	2.81	2.22	8.61 [c]	1842
	3421.932	8.06	3.62	2.08	2.81	2.22	8.61 [c]	1951
	3422.014	8.06	3.62	2.08	2.81	2.22	8.61 [c]	2028
12	3422.102	4.13	2.94	1.68	2.43	1.57	17.11 [c]	1183

	3421.932	4.13	2.94	1.68	2.43	1.57	17.11 [c]	1330
	3422.014	4.13	2.94	1.68	2.43	1.57	17.11 [c]	1287
13	3422.102	4.13	2.94	1.68	2.43	2.25	17.11 [c]	2115
	3421.932	4.13	2.94	1.68	2.43	2.25	17.11 [c]	2039
	3422.014	4.13	2.94	1.68	2.43	2.25	17.11 [c]	2173
14	3422.102	10.12	3.62	2.08	3.52	3.62	8.68 [c]	3277
	3421.932	10.12	3.62	2.08	3.52	3.62	8.68 [c]	3375
	3422.014	10.12	3.62	2.08	3.52	3.62	8.68 [c]	3140
15	3422.102	10.12	3.62	2.08	3.52	1.94	8.68 [c]	1587
	3421.932	10.12	3.62	2.08	3.52	1.94	8.68 [c]	1631
	3422.014	10.12	3.62	2.08	3.52	1.94	8.68 [c]	1710
16	3422.102	8.21	2.96	1.69	3.95	2.96	17.36 [c]	2529
	3421.932	8.21	2.96	1.69	3.95	2.96	17.36 [c]	2668
	3422.014	8.21	2.96	1.69	3.95	2.96	17.36 [c]	2775
17	3422.102	8.21	2.96	1.69	3.95	1.58	17.36 [c]	1317
	3421.932	8.21	2.96	1.69	3.95	1.58	17.36 [c]	1460
	3422.014	8.21	2.96	1.69	3.95	1.58	17.36 [c]	1341

[a] In molecule cm^{-3}.

[b] The initial concentration of Cl atoms is obtained from the measured time traces of HO_2 by performing kinetic model fitting under the same experimental conditions but without NO.

[c] N_2 is used as the buffer gas.

Supplementary References

1. Tang, Y., Tyndall, G. S. & Orlando, J. J. Spectroscopic and kinetic properties of HO_2 radicals and the enhancement of the HO_2 self reaction by CH_3OH and H_2O. *J. Phys. Chem. A* **114**, 369–378 (2010).
2. *Hitran database*. http://hitran.org/.
3. Zahniser, M. S., McCurdy, K. E. & Stanton, A. C. Quantitative spectroscopic studies of the hydroperoxo radical: band strength measurements for the ν_1 and ν_2 vibrational bands. *J. Phys. Chem.* **93**, 1065–1070 (1989).
4. Goldman, A. et al. Updated line parameters for OH $X^2\Pi-X^2\Pi$ (ν'',ν') transitions. *J. Quan. Spec. Rad. Trans.* **59**, 453−469 (1998).
5. Burkholder, J. B., Sander, S. P., Abbatt, J., Barker, J. R., Huie, R., Kolb, C. E., Kurylo, M., Orkin, V., Wilmouth, D. & Wine, P. H. Chemical kinetics and photochemical data for use in atmospheric studies. Evaluation No. 15; JPL (2015).

6. Pagsberg, P., Munk, J., Sillesen, A. & Anastasi, C. UV spectrum and kinetics of hydroxymethyl radicals. *Chem. Phys. Lett.* **146**, 375−381 (1988).
7. Meier, U., Grotheer, H. H., Riekert, G. & Just, Th. Study of hydroxyl reactions with methanol and ethanol by laser-induced fluorescence. *Ber. Bunsenges. Phys. Chem.* **89**, 325–327 (1985).
8. Tyndall, G.S., Orlando, J.J., Kegley-Owen, C.S., Wallington, T.J. & Hurley, M. D. Rate coefficients for the reactions of chlorine atoms with methanol and acetaldehyde. *Int J. Chem. Kinet.* **31**, 776–784 (1999).
9. Grotheer, H.-H., Riekert, G., Meier, U. & Just, Th. Kinetics of the reactions of CH_2OH radicals with O_2 and HO_2. *Ber. Bunsenges. Phys. Chem.* **89**, 187–191 (1985).
10. Veyret, B., Lesclaux, R., Rayez, M-T., Rayez, J-C., Cox, R.A. & Moortgat, G.K. Kinetics and mechanism of the photooxidation of formaldehyde. 1. Flash photolysis study. *J. Phys. Chem.* **93**, 2368–2374 (1989).
11. Pagsberg, P., Munk, J., Anastasi, C. & Simpson, V.J. Reaction of CH_2OH with O_2, NO, and NO_2 at room temperature. *J. Phys. Chem.* **93**, 5162–5165 (1989).
